# Circular Economy Applied to Sludge Minimization: The STAR Project

**DOI:** 10.3390/membranes15010015

**Published:** 2025-01-09

**Authors:** Maria Cristina Collivignarelli, Stefano Bellazzi, Alessandro Abbà

**Affiliations:** 1 Department of Civil Engineering and Architecture, University of Pavia, Via Ferrata 3, 27100 Pavia, Italy; mcristina.collivignarelli@unipv.it; 2 Interdepartmental Centre for Water Research, University of Pavia, Via Ferrata 3, 27100 Pavia, Italy; 3 Department of Civil, Environmental, Architectural Engineering and Mathematics, University of Brescia, Via Branze 43, 25123 Brescia, Italy; alessandro.abba@unibs.it

**Keywords:** sludge minimization, resource recovery, thermophilic membrane reactor, respirometric tests

## Abstract

The management of biological sludge from wastewater treatment plants (WWTPs) poses a significant environmental challenge due to increasing sludge production and the presence of emerging pollutants. This study investigates an innovative solution by integrating a thermophilic aerobic membrane reactor (TAMR) into the sludge treatment line of a medium-size WWTP, aiming to minimize biological sludge output while enhancing resource recovery. The study involved a six-month monitoring of an industrial-scale TAMR system, assessing the reduction in volatile solids (VSs) in thickened sludge and evaluating the compatibility of TAMR residues with conventional activated sludge (CAS) systems. The TAMR unit, which achieved up to a 90% reduction in VSs, was combined with traditional CAS processes, forming the STAR (Sludge Treatment and Advanced Recycling) configuration. This configuration reduced sludge output to just 10% of conventional levels while enabling the recirculation of nutrient-rich liquid effluents. Both batch and continuous respirometric tests demonstrated the biological treatability of TAMR residues, highlighting their potential reuse as external carbon sources and their positive impact on CAS system performance. The findings suggest that integrating mesophilic and thermophilic systems can significantly improve sludge management efficiency, lowering both operating costs and environmental impacts.

## 1. Introduction

The management of biological sludge from wastewater treatment processes represents one of the most challenging issues in the environmental sector. Sludge production is increasing due to the enhanced efficiency of wastewater treatment plants (WWTPs), but the situation is made more difficult by the appearance of emerging pollutants, many of which are of anthropogenic origin, and the stricter regulations [1]. These factors are pushing toward sludge minimization solutions, such as thermal oxidation and incineration techniques [2]. However, these technologies have a significant environmental drawback in terms of CO_2_ emissions from sludge combustion [3] and they also result in the loss of valuable resources such as carbon, nitrogen, and phosphorus, which are abundantly present in sludge [4].

The introduction of stricter regulations for the discharge of effluents from urban WWTPs, as established by the European Directive 91/271/EEC [5] (European Commission, 1991) and its subsequent amendments, has led to an increase in the production of sewage sludge, while worsening its qualitative characteristics [6]. Directive 2018/851/EC [7] (European Commission, 2018) established a hierarchy for waste management, which is also applicable to sewage sludge. This hierarchy prioritizes: (i) waste prevention and minimization; (ii) material recovery and reuse of residual sludge; (iii) energy recovery, and; (iv) the safe disposal of residues [8]. Building on this framework, the European Directive 2024/2019 (https://eur-lex.europa.eu/legal-content/EN/TXT/?uri=OJ:L_202403019, accessed on 22 December 2024) recently adopted new rules to further enhance urban wastewater treatment, updating the 1991 directive to address contemporary environmental and public health challenges. Key measures include extending treatment coverage to smaller urban areas, reducing pollutants, promoting renewable energy use, and implementing real-time monitoring. These innovations aim to enhance sustainability, transparency, and circular economy in water resource management, aligning with the European Green Deal. The adoption will occur gradually over the next few years.

The choice of the most suitable technology to reduce and prevent sludge production must take into account both technical and economic factors, carefully weighing the pros and cons [9]. Minimizing biological sludge production is crucial, not only to comply with the regulations but also for the significant benefits it provides. One key advantage is the reduction in operating costs, as sludge management can account for up to 50% of a WWTP operating expense [10]. Beyond economic costs, the environmental impact of sludge treatment, transport, and disposal must also be considered [11].

A major challenge in urban water management is transforming traditional WWTPs into water resource recovery facilities (WRRFs) [12]. This transition requires preventing and minimizing sludge production through two primary strategies: (i) implementing processes that minimize residual sludge during water treatment, or (ii) applying in situ treatments to further reduce sludge production [13].

An effective approach involves combining mesophilic biological treatments, such as conventional activated sludge (CAS) technology, with thermophilic treatments in the sludge line of conventional WWTPs [14]. A thermophilic fluidized bed reactor, utilizing membrane filtration, can achieve up to a 90% reduction in volatile solids (VSs) in biological sludge. However, thermophilic sludge lacks optimal mechanical filtration and settling properties [15]. Therefore, it is crucial to investigate the synergistic effects of both thermophilic and mesophilic degradative treatments in both directions.

This study evaluated the start-up and steady-state operation of an industrial-scale thermophilic membrane treatment applied to pre-thickened sludge from a medium-size WWTP (serving 80,000 population equivalents) over a six-month period. Additionally, the mesophilic treatability of the liquid residues and treated sludge was analyzed, demonstrating an integrated approach to the management of sewage sludge, direct-in-line sludge, and treatment residues recirculated back to the water line. The novelty of this work lies in its focus on the first and only full-scale thermophilic aerobic membrane plant in Italy, designed to minimize biological sludge production. Beyond evaluating the system’s performance in reducing VSs, the research also explored its compatibility with a conventional activated sludge treatment plant.

## 2. Material and Methods

### 2.1. Thermophilic Aerobic Membrane Reactor: TAMR Description

The Thermophilic Aerobic Membrane Reactor (TAMR) technology combines thermophilic aerobic digestion using pure oxygen, which is directly injected inline through a Venturi device, with ultrafiltration via ceramic membranes. The crossflow ultrafiltration system consists of three vessels with 99 membranes each, featuring 23 channels, a cut-off of 300 kDa, and a pore size of 10 nm. The biological reactor has a volume of 200 m^3^ and can treat 25 m^3^ d^−1^ of thickened biological sludge.

TAMR is integrated into the sludge treatment line to significantly reduce the amount of biological sludge produced by a wastewater treatment plant. The primary goal is to minimize biological sludge while producing a sanitized and stabilized product suitable for agricultural reuse [16]. This product undergoes upstream treatment in an energy recovery process, such as digestion, to hydrolyze and break down high-molecular-weight compounds that typically hinder digestion efficiency.

### 2.2. Analytical Methods

To analyze the thickened biological sludge feed samples and the thermophilic sludge inside the biological reactor, the regulations established by the Lombardy Region (Italy) must be followed [17]. The pH, TS, and VSs were measured in accordance with APAT-IRSA-CNR 2060 (2003) [18], UNI EN 14346 (2007) [19], and UNI EN 15169 (2007) [20], respectively. Organic carbon was studied according to CNR-IRSA 5 (1988) [21]. COD was monitored according to the Standard Methods for the Examination of Water and Wastewater (APHA, 2012) [16,22]. The dissolved oxygen (O_2_) and the temperature in the reactor were measured by a submerged probe (Endress + Hauser Oxymax W COS31).

### 2.3. Characteristics of Fed Thickened Biological Sludge

The main characteristics of the substrate (thickened biological sludge) fed into the TAMR are shown in Table 1.

### 2.4. Oxygen Uptake Rate (OUR) Tests

The OUR test measures dissolved oxygen (O_2_) consumption over time by biomass in each sample [23]. Endogenous OUR reflects oxygen used solely for biomass respiration, while exogenous OUR includes additional oxygen consumed for oxidizing biodegradable organic matter or nitrogen compounds when an external substrate is present. O_2_ concentration was measured by WTW multi-parameter portable meter MultiLine^®^ Multi 3510 IDS thanks to WTW Optical IDS dissolved oxygen sensors FO2^®^ 925 (Xylem Analytics Germany Sales GmbH & Co, Mainz, Germany) (called O_2_ probe in the next sections). The measured O_2_ concentration was transferred to a PC via USB connection and the MultiLab^®^ Importer for data acquisition via Excel^®^ software (version number 2409) was used.

Temperature and pH were measured using the probe WTW-IDS, Model SenTix^®^ 940 (Xylem Analytics Germany Sales GmbH & Co, Mainz, Germany).

#### 2.4.1. Batch Tests

The experimental setup involves a flask, magnetic stirrer with a bar, dissolved oxygen (O_2_) probe, computer, aerator, porous stone, and sealing film. Initially, the VSs in the biomass (gVS L^−1^) are measured. The biomass is then placed in a flask on a magnetic stirrer to ensure continuous mixing. A porous stone, connected to the aerator, and the O_2_ probe are immersed in the biomass to monitor oxygen levels. The biomass is aerated until oxygen saturation is reached, then aeration is stopped. For exogenous tests, the substrate is added after aeration stops, and the flask is sealed with parafilm to prevent air exchange. Data are recorded every 5 s. At the end of each test, the O_2_ trend over time is plotted, and the slope of the linear regression line in the central curve section is calculated. Specific Oxygen Uptake Rate (sOUR) is determined by dividing this slope by the VS concentration, isolating oxygen consumption from the degradation of organic matter in the substrate alone. If needed, the OUR is normalized to 20 °C using a temperature adjustment formula. Endogenous tests (400 mL biomass) measure endogenous respiration, while exogenous tests (400 mL biomass + 400 mL substrate) analyze exogenous respiration. Each test is repeated for accuracy, with standard deviation and 95% confidence intervals calculated for each condition [24].

#### 2.4.2. Continuous Tests

The experimental apparatus used to carry out continuous OUR is the same as that presented for batch OUR tests of the mixed liquor present inside the test and therefore to perpetuate the respirometric analysis over time [25]. Continuous OUR tests generated a respirogram, showing the trend of OUR over time [26]. Oxygen consumption was monitored to assess both endogenous and exogenous respiration phases after adding a carbon substrate. The test continued until the substrate was nearly depleted when exogenous sOUR matched the endogenous rate. The continuous OUR test aimed to evaluate medium- to long-term toxic or inhibitory effects of the substrate on biomass, and to measure organic matter removal by calculating total oxygen consumption across exogenous and endogenous phases. These tests were conducted in a lab-scale reactor containing 400 mL of biomass and 400 mL of substrate, composed of wastewater typical for the plant [27], and some thermophilic sludge [28] to test treatability in the mesophilic phase. The reactor was sealed to prevent oxygen and moisture loss, with aeration maintained at 2–5 mg L^−1^ using an electronically controlled aeration system linked to software on a PC, which continuously recorded O_2_ data. Tests ran from 8 h to 5 days, producing O_2_ consumption curves reflecting alternating aeration and non-aeration. Additional sOUR values were calculated, and the respirogram was plotted. Total oxygen consumption was quantified by integrating the exogenous curve and subtracting the estimated area related to endogenous respiration (Equation (1)).

(1) $$\int_{t_{start}}^{t_{end}}sOUR(t)dt-\int_{t_{start}}^{t_{end}}sOUR_{endo(t)dt}$$

### 2.5. Rheological Measurements

In the literature, several models are available to interpolate the shear stress–shear rate relationship, based on experimental measurements, to obtain the corresponding flow curve. When dealing with thermophilic biological sludge from a membrane system, the Herschel–Bulkley model (Equation (2)) is often well suited for fitting experimental data from these sludge samples, which are characterized by a high solid concentration.

(2) $$\tau =\tau_{0}^{HB}+k{\dot{\gamma }}^{n}$$

where **τ** (Pa) is the shear stress, and (**γ**) represents the shear rate. **τ_0_** (Pa) is the yield shear stress, which quantifies the stress the fluid must experience before it yields and begins to flow. The constants ***k*** and ***n*** are specific to the fluid: ***k*** indicates the fluid’s consistency, with higher values corresponding to more viscous fluids, while ***n***, typically less than 1, reflects the non-Newtonian behavior. The further ***n*** deviates from 1, the more the fluid’s characteristics differ from those of a Newtonian fluid [29].

## 3. Results

### 3.1. Start-Up Phase of the TAMR System

**Figure 1:**
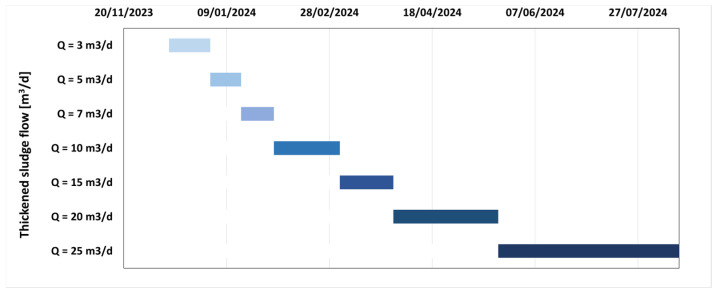
Increase in the flow rate treated by the TAMR. The shades of blue darken until steady state conditions are reached.

The start-up phase of the TAMR system lasted approximately three months, during which the focus was on achieving the desired operating conditions and reaching a steady state to ensure maximum process reliability and robustness. Figure 1 presents a Gantt chart illustrating the strategy for gradually increasing the flow rates entering the thermophilic reactor. The fully operational conditions were achieved by following the indications coming from various experimental works, some already published in international scientific journals, in which the optimal HRT (hydraulic retention time) was identified in 10 days [16].

### 3.2. TAMR Plant Monitoring

**Figure 2:**
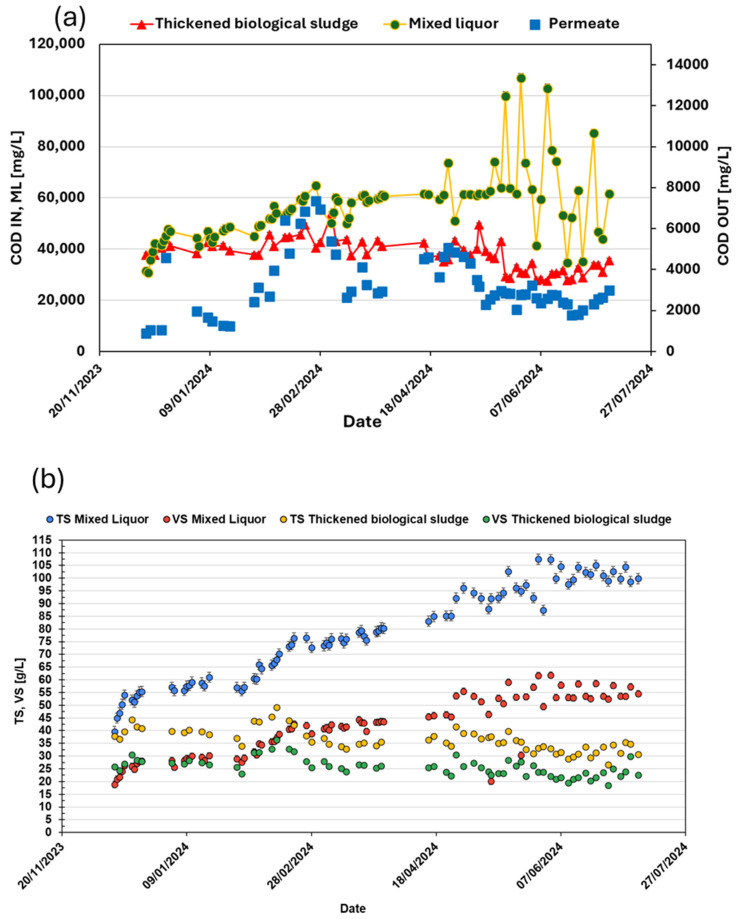
Characteristics of the biological sludge entering the TAMR and within the reactor itself. (**a**): a trend of COD concentrations at the input (thickened biological sludge), output (liquid residue), and within the biological reactor (mixed liquor). (**b**): trend of total solids and volatile solids concentrations both entering the TAMR and inside the reactor.

The trend of the biological sludge characteristics is shown in Figure 2, including total solids and volatile solids, as well as the related thermophilic chemical oxygen demand (COD) concentrations observed during the start-up phase and the monitoring period when the technology reached a steady state.

**Figure 3:**
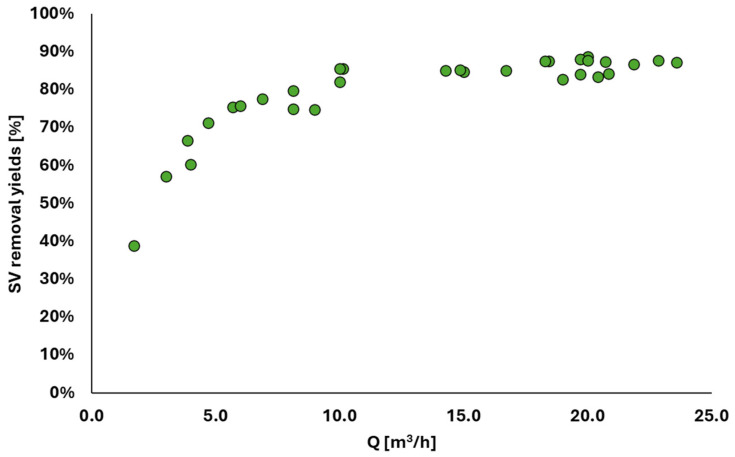
Volatile solid (VS) removal efficiency of the TAMR as a function of the daily flow rates treated.

**Figure 4:**
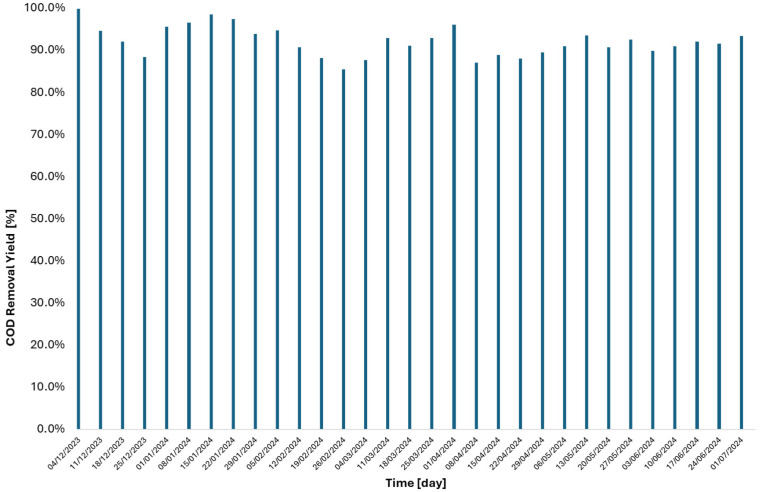
COD removal efficiency of the TAMR calculated as the difference between the inlet-thickened sludge load and the extracted liquid residue.

The removal efficiency of TAMR technology applied to the organic content of biological sludge, specifically the VSs, is illustrated in Figure 2b. The efficiency was determined by dividing the mass of volatile solids removed by the inlet mass of volatile solids. Figure 3 shows the relationship between VS removal efficiency and the flow rate treated by the technology. As indicated in the graph, the removal efficiency increases with higher flow rates, achieving an average efficiency of 90% for flow rates above 10 m^3^ d^−1^. In Figure 4, the treatment efficiency of the TAMR technology with respect to COD is presented. This parameter is evaluated by calculating the reduction efficiency, which is determined as the percentage of reduction in COD levels between the thickened sludge entering the thermophilic reactor and the liquid effluent exiting it. The results highlight the ability of the TAMR system to significantly reduce COD content, providing insights into its performance in the treatment process. This yield was immediately confirmed to be above 80% even in the start-up phases of the technology.

**Figure 5:**
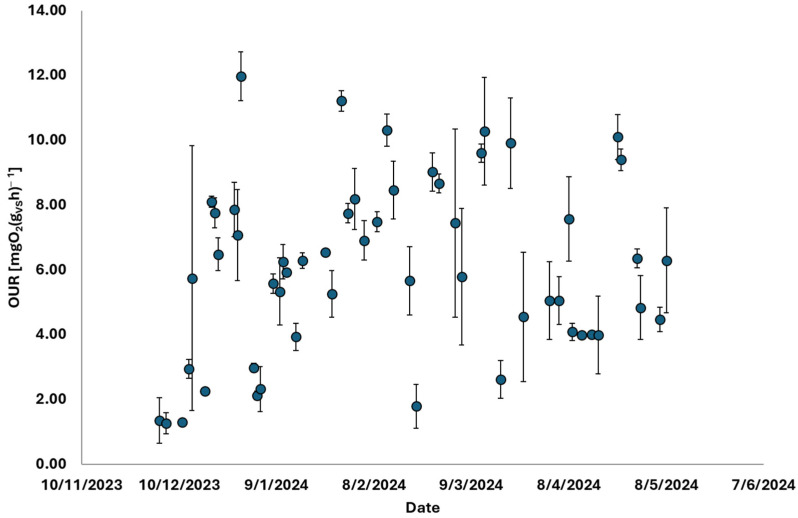
Trend of endogenous OUR (Oxygen Uptake Rate) values carried out on thermophilic biomass to evaluate its health.

The results of the endogenous OUR tests conducted on the thermophilic biomass inside the TAMR, aimed at assessing its health status, are presented in Figure 5. After an initial acclimatization period corresponding to the early start-up phase of the process, the biomass respiration stabilized at an endogenous respiration rate of 8 mgO_2_(gSV·h)^−1^. The error bars displayed in the graph indicate the confidence intervals, determined by performing the tests in duplicate.

**Figure 6:**
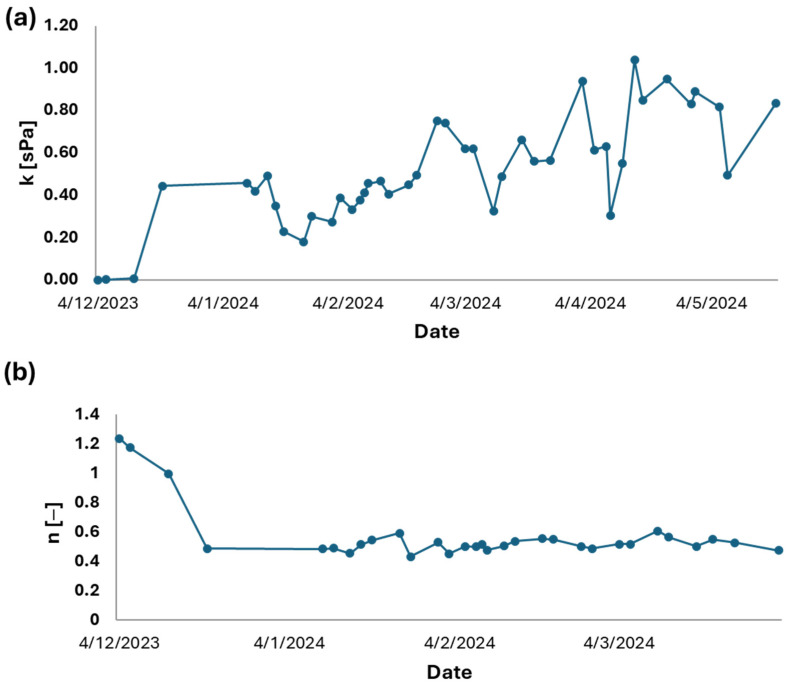
Trend of rheological parameters of thermophilic sludge. (**a**) shows the trend of the k-consistency value of the fluid. (**b**) shows the trend of parameter n, which indicates the distance from Newtonian behavior.

The trends observed over six months of monitoring the rheological parameters of the Herschel–Bulkley model are illustrated in Figure 6, focusing on fluid consistency (K) and the flow index (n), which clearly deviates from Newtonian behavior. After an initial start-up phase—characterized by the acclimatization of the thermophilic biomass and an increase in the concentration of solids within the thermophilic mixed liquor (as confirmed by the solid concentration trend in Figure 2b)—the fluid-mechanical properties inside the thermophilic reactor reached a stable state. This stabilization is particularly significant for the analysis of the ultrafiltration process during the solid–liquid separation phase [30].

### 3.3. TAMR—CAS Compatibility: Evaluation and Recovery of Residues

To evaluate the treatment efficiency of the TAMR system in terms of reducing thickened sewage sludge, both single-section and continuous OUR restitution measurements were conducted on the two residues produced by the treatment process. These residues include the liquid effluent (liquid residue) from the ultrafiltration membranes and a low amount of sludge residue (thermophilic sludge), whose extraction helps to maintain the solids concentration in the biological reactor within the desired range. This integrated approach, which goes beyond the simple calculation of performance yields, aims to analyze the respiration of a conventional activated sludge system in which the two-treatment residues have been recirculated, not stopping at simple process yields but studying the process from start to finish, including the treatment residues [31]. To evaluate the reduction in biological sludge in a wastewater treatment plant through the implementation of the TAMR system, this study employed an integrated approach based on the STAR (Sludge Treatment and Advanced Recycling) scheme reported in the discussion paragraph.

**Figure 7:**
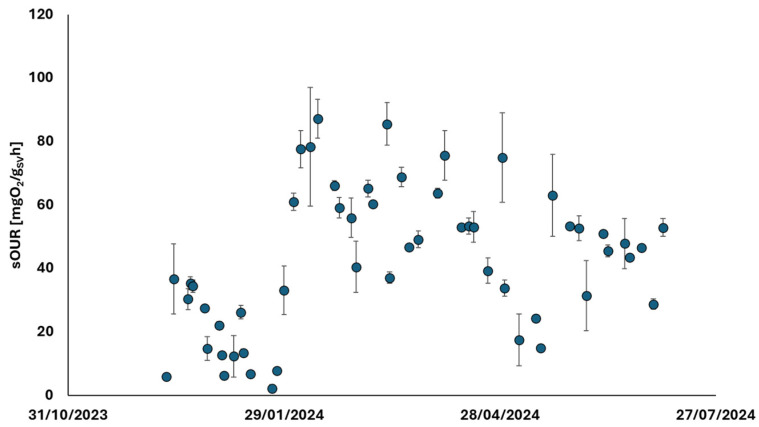
Trend of exogenous OUR (Oxygen Uptake Rate) values carried out by placing the liquid residue of the TAMR (liquid residue) in contact with traditional mesophilic biomass with activated sludge to evaluate its biological treatability.

The progression of OUR tests, shown in Figure 7, involved placing the liquid residue from the TAMR treatment in contact with mesophilic biomass in the activated sludge process. After an initial phase, during which the liquid residue quality was transformed—corresponding to the plant’s start-up period—the respiration rates indicated a high level of biological treatability for this residue. This finding suggests that the treatment residue can be effectively managed by recirculating it within traditional oxidation tanks, where its organic content could significantly enhance biological kinetics.

After assessing the treatability of the liquid residue via OUR batch tests, the treatability of the residual sludge was assessed. This was achieved by performing continuous respirometric tests. In these tests, a portion of thermophilic purge sludge was added to a continuous OUR test that measured the respiration of the biomass from a conventional activated sludge exposed to its usual influent wastewater. The influent was supplemented with the added sludge portion. These tests, lasting an average of 8 h, simulated the HRT observed in full-scale plant oxidation tanks. Furthermore, incremental increases in both solid and organic loading were applied to progressively enhance the tested sludge portion within the experiment. To assess the potential inhibitory effects of the purge sludge portion, respiration rates were monitored over time, and the total oxygen consumption throughout the test was calculated. This was carried out by computing the area under the respiration curve and comparing it with the COD portion introduced by the sludge inoculum.

The results of these tests are shown in Table 2, detailing the tested load steps, the TS steps, the dosed COD amount, and the total oxygen consumption observed throughout the test duration.

## 4. Discussion

The research focused on the introduction of thermophilic membrane technology, aiming to minimize sludge production. This was monitored throughout the start-up phase, observing several key parameters and trends. Initially, attention was given to the gradual ramp-up of treated flow rates. The system was progressively scaled up until it reached a stable operation at a flow rate of 25 m^3^ d^−1^. During this phase, the concentration of TS within the system was maintained at 35 g L^−1^. In addition, several chemical parameters were closely tracked, such as COD, TS, and VSs. Measurements were taken at three critical points: the inlet, the outlet, and within the biological reactor itself. This allowed for a comprehensive understanding of how the system was handling and treating the incoming waste. The biological health of the system was also a significant focus. Advanced analytical methods were employed to ensure the proper functioning of the biomass. Among these methods, endogenous OUR tests played a central role [32]. These tests were conducted to measure the biomass respiration rates, a key indicator of biological activity and system stability. After an initial acclimatization phase, the biomass reached a steady state, with respiration rates stabilizing at 7 mg O_2_ gVS^−1^·h^−1^. This indicated that the system had successfully adapted to the new operational conditions. In addition to biological assessments, the study also included physical evaluations, specifically rheological measurements during the ultrafiltration phase. These measurements were carried out to monitor the performance of ceramic membranes used in the system. The stability and efficiency of these membranes were critical to the overall success of the TAMR system in reducing sludge production. The integrated approach not only assessed the direct outcomes of the thermophilic membrane technology but also evaluated the treatment of by-products generated by the system. Two types of residues were examined: the liquid residue (liquid residue) and the sludge. Both batch and continuous respirometric tests were conducted to determine the biodegradability and potential environmental impact of these residues. Notably, the liquid residue showed a particularly promising respiratory response. With an average oxygen uptake rate of 50 mg O_2_ gVS^−1^·h^−1^, the liquid residue demonstrated high biodegradability. This value was reached following the start-up phase in which, as confirmed by Figure 2a, the liquid residue stabilized in terms of the organic substance contained. This suggests that it can be effectively processed in a CAS system, enhancing the overall efficiency of substrate degradation and minimizing the environmental footprint of the WWTP.

In summary, the implementation of the TAMR system in the WWTP successfully reduced biological sludge production. The combination of monitoring treated flow rates, analyzing chemical parameters, assessing biomass health, and evaluating residual biodegradability provided a comprehensive picture of the system’s performance. The encouraging results, particularly the high biodegradability of the liquid residue, suggest that thermophilic membrane technology could be a viable strategy for improving the sustainability of wastewater treatment operations. The literature review further supports its potential for recirculation in pre-denitrification processes as a valuable external carbon source [33]. Continuous respiration tests conducted on the sludge residue demonstrated that recycling 1% of the sludge content from the thermophilic reactor back into the CAS system had no inhibitory effects on the biological activity of the activated sludge. On the contrary, this recirculation strategy contributed to further sludge reduction through enhanced bio-oxidation processes. The results underline the robustness and compatibility of integrating thermophilic and mesophilic treatment stages within a single system. By leveraging this integration, traditional WWTP can achieve significant operational and environmental benefits. The thermophilic reactor not only minimizes sludge volume through effective volatile solid degradation but also produces a nutrient-rich residue that can be recirculated to optimize the mesophilic process. This synergy enhances the overall treatment efficiency, reduces excess sludge production, and improves resource recovery. Such an approach redefines the role of WWTPs, transforming them from conventional waste processors into resource-efficient facilities capable of reducing operational costs, lowering waste disposal burdens, and contributing to a circular economy by reclaiming valuable by-products such as carbon sources for biological processes. This integration offers a sustainable pathway for modernizing WWTP operations while meeting stricter regulatory requirements and environmental goals. The findings emphasize the importance of integrating thermophilic and mesophilic processes, transforming traditional WWTPs into resource-efficient facilities [34]. Future research should focus on long-term operational stability, scaling-up processes, and exploring the potential of integrating other advanced technologies for further sludge minimization and resource recovery.

### 4.1. Economic Aspects of Implementing TAMR in the WWTPs Sludge Line

The economic aspects of the proposed technology are discussed in this section. The following are the main elements that influence the process’s economic efficiency: (i) the cost of liquid oxygen; (ii) the cost of electricity; (iii) the thickening degree of the fed sludge.

The organic load in the feed, represented by COD, is closely correlated with the oxygen expenses. The treated biological sludge’s COD content is roughly 10 kgCOD per 1% of TS, and its oxygen consumption rate is roughly 1 kgO_2_/kgCOD.

Electricity costs are inversely correlated with the feed sludge’s degree of thickening and directly correlated with the feed volume (measured in m^3^/h). For biological sludge with 3% of TS in the feed, the average consumption is 65 kWh/m^3^.

Assuming the following costs for the utilities:

- € 0.12/kWh for electricity;
- € 0.10/kgO_2_ for liquid oxygen;

The specific treatment cost, referred to sludge at 18% TS obtained through conventional mechanical dewatering, is between € 70–80/t. This value is much lower than the current market prices of about € 250/t for disposal via incineration, which includes dewatering and transportation. Lastly, the system’s high level of automation allows for remote operation without the need for specialized staff to oversee it.

### 4.2. Sludge Treatment and Advanced Recycling: STAR

**Figure 8:**
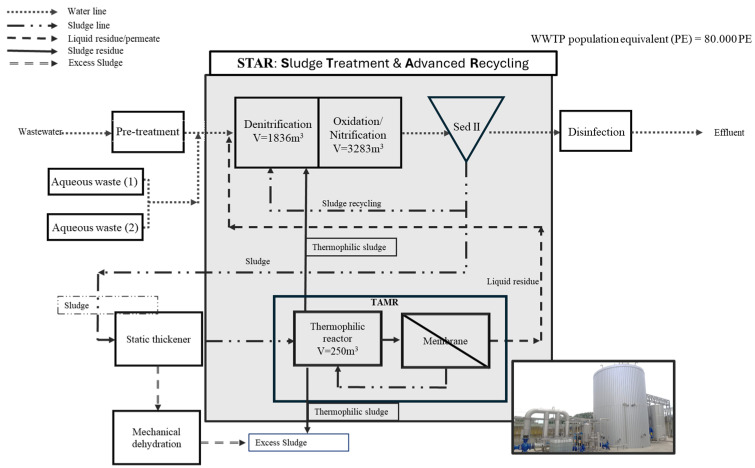
Block diagram of the treatment platform designed for the application of the STAR system. Volumes involved and number of equivalent populations treated.

The STAR framework, as illustrated in Figure 8, represents an innovative approach to integrated sludge treatment in wastewater treatment plants (WWTPs). This scheme combines a CAS with a TAMR for thickened sludge treatment, specifically applied in a WWTP designed to manage a flow rate of 7000 m^3^/d and serve 80,000 population equivalents. The integration of the TAMR unit offers significant benefits, including a substantial reduction in excess sludge, with VS reductions averaging 90%, as confirmed through a six-month experimental phase. This performance was complemented by respiration tests under thermophilic conditions and rheological analyses, which demonstrated stable biomass viability and fluid mechanical properties. Furthermore, respirometric tests carried out over durations longer than 90 h confirmed that the recirculation of nutrient-rich TAMR residues (both liquid and sludge) into the mesophilic unit served as an external carbon source, improving denitrification and overall process performance without medium-term inhibitory effects. In addition to improving resource recovery by recycling effluents as a nutrient supply in the denitrification stage, the suggested STAR design strikes a synergistic balance between mesophilic and thermophilic systems, allowing a worldwide sludge reduction to just 10% of the solids generally produced. For contemporary WWTPs, this integrated approach offers a reliable and scalable solution that supports sustainable resource management and sludge minimization.

## 5. Conclusions

This study successfully demonstrated the potential of integrating a TAMR into a traditional wastewater treatment plant to minimize biological sludge production. The implementation of TAMR technology, as outlined in the STAR scheme, resulted in a significant reduction in excess sludge, achieving an average volatile solid removal efficiency of 90% at steady-state conditions. Key observations during the study included a stable biological performance of the thermophilic biomass after an acclimatization phase, with consistent endogenous respiration rates and favorable rheological properties that support effective ultrafiltration. The evaluation of treatment residues offered valuable insights into the feasibility of residue recirculation. The liquid effluent showed high biological treatability, suggesting its potential to be reintegrated into conventional activated sludge systems to enhance biodegradation and serve as an effective carbon source. Continuous respirometric tests confirmed that the sludge residue from TAMR, when recirculated in small proportions, did not inhibit the performance of mesophilic-activated sludge, enabling further sludge reduction through bio-oxidation. Overall, the TAMR system represents a promising solution for reducing sludge production in WWTPs, providing both environmental and economic benefits.

## Figures and Tables

**Table 1:** Physical–chemical parameters of fed thickened biological sludge. TS: total solids; VSs: volatile solids; n: number of data.

Parameter	Values
COD [mg L^−1^]	20,000–50,000 ± 6320 [n = 80]
pH [-]	5–6 ± 0.9 [n = 80]
TS [g L^−1^]	30–50 ± 2.5 [n = 80]
VSs [g L^−1^]	20–40 ± 2.5 [n = 80]

**Table 2:** Results of continuous respirometric tests for the evaluation of the biological treatability of thermophilic sludge residue. The table details the loading steps used in terms of mL dosed, the quantity of total solids characteristic of the sludge purge tested, the oxygen consumption during the entire duration of the test, the dosed COD, and the percentage of COD removed.

Recirculated Sludge [mL]	TSin [mgTS]	ΔO_2_ Total [mgO_2_ gSV^−1^]	COD Tested [mgCOD]	ΔO_2_ Exogenous [mgO_2_]	COD Removed [%]
1	54	57	205	159	78
2	115	59	256	132	51
2	114	88	257	146	57
3	167	109	306	226	74
4	264	103	388	216	56
3	228	103	334	196	59

## Data Availability

The raw data supporting the conclusions of this article will be made available by the authors on request.
